# Chemical and radioanalytical investigations of ^106^Ru-containing air filters from Vienna in fall 2017: searching for stable element anomalies

**DOI:** 10.1007/s10967-018-6132-6

**Published:** 2018-09-01

**Authors:** Dorian Zok, Johannes H. Sterba, Georg Steinhauser

**Affiliations:** 1 Institute of Radioecology and Radiation Protection, Leibniz Universität Hannover, Herrenhäuser Str. 2, 30419 Hannover, Germany; 2 Atominstitut, TU Wien, Stadionallee 2, 1020 Vienna, Austria

**Keywords:** Neutron activation analysis, Radioruthenium, ^106^Ru, Environmental air filter, Stable element anomalies

## Abstract

Related to the recent nuclear release of radioactive ruthenium isotopes in fall 2017, we analyzed air filters from Vienna for irregularities in the (stable) elemental composition of particulate matter from this period. Methods were SEM/EDXS and INAA. For comparison, a reference filter from 2007 and blank filters were used. The chemical fingerprint encompassed 28 elements. The results show no indication for a considerable change in the elemental composition of the suspended matter. For example, no anomalies in the abundance of platinum group elements were found. The results suggest that the release of ^106^Ru had not been accompanied by a release of detectable amounts of (activatable) stable elements.

## Introduction

The release of radioactive materials into the environment is inherently associated with great public concern. The radioactive fallout from atmospheric nuclear explosions in the 20th century has been largest contributor to anthropogenic radionuclides in the environment. At the height of the cold war in the early 1960s, global fallout reached a magnitude that was no longer irrelevant for the public health. These concerns ultimately triggered diplomatic attempts to ban atmospheric nuclear tests by establishing the Partial Test Ban Treaty (PTBT; sometimes also referred to as Limited Test Ban Treaty, LTBT), which was opened for signature in 1963. In 1996, the Comprehensive Nuclear-Test-Ban Treaty (CTBT) opened for signature [1], aiming to terminate nuclear testing in any environment. For the verification of the CTBT, the international monitoring system (IMS) was installed, designed to detect any violations of the CTBT by geophysical and radionuclide monitoring of the globe. The establishment of the IMS coincided with increased attempts of national governmental efforts to set up monitoring networks that would allow the detection and public risk assessment of undeclared nuclear releases such as the Windscale or Kyshtym accidents in 1957 [2] or the Chernobyl accident in 1986.

Two unusual incidents of radionuclide releases occurred in 2017. In January/February of that year, an unusually long episode of ^131^I was observed [3]. In fall 2017, European monitoring stations reported an unusual and unprecedented detection of radioruthenium in air [4]. Rapid gamma measurements revealed the presence of radioactive ^106^Ru (*T*_½_ = 373.6 days), and in some stations also ^103^Ru (*T*_½_ = 39.2 days) in air. Relatively little is known about the release at this point. However, several monitoring stations reported of futile attempts to detect other radionuclides in addition with the radioruthenium. This indicates that the source of the radioruthenium was probably not an accident of a nuclear reactor. Until today, the source remains uncertain and intensely debated [4]. Although ^103^Ru may be produced by neutron activation of stable ^102^Ru, both radioruthenium nuclides are prominent fission products. The fission yield, however, largely depends on the type of fissile material, as ^239^Pu based nuclear fuel produces ^106^Ru at a higher yield than ^235^U. For ^103^Ru, this difference in the yield is not so pronounced. More on the nuclear background of the production can be found elsewhere [5].

In the present study, we aim at the analysis of stable element analysis in order to establish a chemical fingerprint of the airborne particulate matter at the time of the release. We are interested in the elemental composition of the particulate matter as the released radioruthenium may have been associated with some anomalous stable elements that went unnoticed so far. Such anomalies may be represented on the ^106^Ru containing air filters. Therefore, we performed instrumental neutron activations analysis (INAA) in Vienna and electron microscopy to elucidate the chemical composition and morphology of the particulate matter contained within the filter materials.

## Experimental

### Location and sampling

The high-volume air filter system used filters made of polypropylene (PP) and was stationed in Vienna, Austria during the ^106^Ru episode. Collection time was nearly 140 h, namely from the 28 Sep. 2017 12:24 to 04 Oct. 2017 8:14. A total volume of 94,444 m^3^ air passed through the PP filter during this time, during which the maximum of the ^106^Ru plume passed Vienna. After collection, the filter was pressed to a round sample with 5.0 cm diameter, a height of 0.55 cm and a mass of 9.7 g. The round filter was measured by gamma spectrometry and then split in half (5.4 g). This segment was used in this study. The total activities collected on the filter were 546 Bq ^7^Be, 461 Bq ^212^Pb, 0.66 Bq ^103^Ru, and 2030 Bq ^106^Ru, respectively. Activities are decay-corrected to 04.10.2017, 08:30. Radioberyllium (^7^Be) and radiolead (^212^Pb) are typical naturally occurring radionuclides, which are continuously produced in the earth’s higher atmosphere (^7^Be) or by decay of primordial ^232^Th, respectively. Their presence in the air filter hence comes expectedly [6, 7].

### Instrumental neutron activation analysis

For INAA, pellets were punched from the PP filter using a punch press with 0.5 cm diameter. Each pellet has a mass of around 80 mg. Three unused PP filter pellets (filter background), two reference PP filter pellets from 2007 (air particulate matter background) and three ^106^Ru-containing PP filter pellets were put in polyethylene (PE) vials. In addition, approx. 35 mg of five certified reference materials were used for a quantitative analysis of the elemental composition. The following reference materials were used: NIST SRM 1633b/Coal Fly Ash (CFA), NIST SRM 2702/Inorganics in Marine Sediment (IMS), NIST SRM 173c/Titanium Alloy (TIA), MC Rhyolite GBW 07113 (GBW) and BCR No. 142/Light Sandy Soil (LSS). An overview of the activation products and the reference materials used for quantification is given in Table 1. This table also outlines, which reference materials have been used for quantification (partly, mean values of various reference materials were used.

**Table 1:** Elements, activation products, half-lives, gamma energies, and reference materials used for quantitative analysis

Element	Activation product	Half-life	Gamma energy (keV)	CFA	IMS	TIA	GBW	LSS
Short time activation analysis								
Al	^28^Al	2.25 min	1778	x	x	x	x	x
Ca	^49^Ca	8.72 min	3084	x			x	x
Ti	^51^Ti	5.8 min	320	x	x	x		x
V	^52^V	3.75 min	1434	x	x	x	x	x
Mn	^56^Mn	2.58 h	1810	x	x	x	x	x
Dy	^165^Dy	2.35 h	95	x			x	x
Middle time activation analysis (5 days decay)								
Na	^24^Na	15.0 h	2754	x	x		x	x
K	^42^K	12.4 h	1525	x	x		x	x
As	^76^As	26.4 h	559	x	x		x	x
La	^140^La	40.3 h	1596	x	x		x	x
Sm	^153^Sm	46.3 h	103	x	x		x	x
Lu	^177^Lu	6.7 days	208	x			x	x
U	^239^Np*	56.6 h	278	x	x		x	x
Long time activation analysis (21 days decay time)								
Sc	^46^Sc	83.8 days	1121	x	x		x	x
Cr	^51^Cr	27.7 days	320	x	x	x	x	x
Fe	^59^Fe	44.5 days	1099	x	x	x	x	x
Co	^60^Co	5.27 days	1173	x	x	x	x	x
Zn	^65^Zn	244.3 days	1116	x	x		x	x
Rb	^86^Rb	18.6 days	1077	x	x		x	x
Zr	^95^Zr	64.0 days	757	x		x	x	x
Ru	^103^Ru	39.2 days	497			x		
Sb	^124^Sb	60.2 days	1691	x	x		x	x
Ba	^131^Ba	11.5 days	496	x	x		x	x
Cs	^134^Cs	2.07 days	796	x	x		x	x
Ce	^141^Ce	32.5 days	145	x	x		x	x
Nd	^147^Nd	11.0 days	531	x	x		x	x
Eu	^152^Eu	13.5 days	1408	x			x	x
Tb	^160^Tb	72.3 days	879	x			x	x
Yb	^169^Yb	32.0 days	177	x			x	x
Lu	^177^Lu	6.7 days	208	x			x	x
Hf	^181^Hf	42.4 days	482	x			x	x
Ta	^182^Ta	114.4 days	1221	x			x	x
Th	^233^Pa*	27.0 days	312	x	x		x	x

x certified reference value used for quantitative analysis

*^233^Pa and ^239^Np are produced by β-decay of the activation products of ^232^Th and ^238^U, respectively. Neutron capture forms short-lived ^233^Th (*T*_1/2_ = 22.3 min) and ^239^U (*T*_1/2_ = 23.5 min), respectively

A short-time (2 min) and a long-time irradiation (32 h) was performed at the 250 kW TRIGA Mark II research reactor of the Atominstitut in Vienna, Austria. The short-time irradiation was performed with the pneumatic sample transfer system into the F-ring of the reactor (thermal neutron flux density 2 × 10^12^ cm^−2^ s^−1^). The long term irradiation took place in a dry irradiation tube in the graphite reflector, with a neutron flux density of about 1 × 10^12^ cm^−2^ s^−1^.

For the analysis of short-lived, activatable radionuclides such as ^28^Al, ^49^Ca, ^51^Ti, ^52^V, ^56^Mn, and ^156^Dy, the sample vials were transferred into the irradiation position by means of a pneumatic transfer system (neutron flux density of approx. 3 × 10^12^ cm^−2^ s^−1^). After 2 min irradiation, sample vials were cleaned on the outside (adding up to 5 min cooling time) and measured immediately by gamma spectrometry with a measurement time of 5 min, yielding the activities of ^28^Al, ^49^Ca, ^51^Ti, and ^52^V. Following a 3 h decay, another measurement was performed with a measurement time of 12 min, yielding the activities of ^56^Mn and ^156^Dy. Gamma spectrometry was performed with a 151 cm^3^ HPGe-detector (1.8 keV resolution at the 1332 keV ^60^Co peak, 50.1% relative efficiency), and a multi-channel analyzer with a preloaded digital filter and loss-free counting system [8, 9].

After the short-time irradiation, samples were irradiated for 32 h in the dry irradiation tube of the TRIGA Mk II in Vienna. After a decay time of 5 days, the medium-lived activation products ^24^Na, ^42^K, ^76^As, ^140^La, and ^239^Np (U) were measured. After 21 days of cooling, the long-lived activation products ^46^Sc, ^51^Cr, ^59^Fe, ^60^Co, ^65^Zn, ^86^Rb, ^95^Zr, ^124^Sb, ^131^Ba, ^134^Cs, ^141^Ce, ^147^Nd, ^152^Eu, ^160^Tb, ^169^Yb, ^177^Lu, ^181^Hf, ^182^Ta, and ^233^Pa (Th) were measured. The measurement time was 1800 s and 10,000 s, respectively. All quantifiable elements, including their neutron activation products, their half-life and gamma energy is listed in Table 1. Additionally, it is noted which reference material has a certified value for which element. Further information on the measurement can be found elsewhere [10–12].

### Particle size and main elemental composition

For a microscopic inspection of the particles collected on the filter surface, scanning electron microscopy (SEM) was used. Without further preparation, the surface of the pressed PP filter samples was imaged with the Philips SEM XL30 ESEM, which is coupled with an energy dispersive X-ray spectroscopy (EDXS) system by EDAX. SEM pictures were taken in back-scattered electron mode and a voltage of 20 kV. With this method, an overview of existing particles and their general size was obtained to check for any apparent anomalies. Furthermore, it allows the investigation of the elemental distribution of the particles by using the EDXS.

## Results and discussion

### INAA

**Fig. 1:**
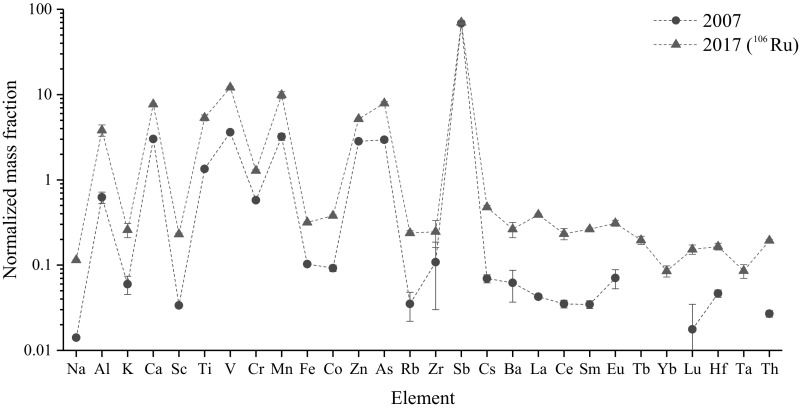
Distribution pattern of elemental mass fractions of two PP filters from Vienna (2007 and 2017, respectively). All values are normalized to the average elemental abundance of the upper continental crust [13]

The results of the INAA are shown in Fig. 1, whereby the mass fractions of the elements are normalized to the content of the upper continental crust [13, 10]. The data points in blue represent the normalized concentrations of the radioactive filter that was in operation during the ^106^Ru episode in 2017. The red data points represent the normalized concentrations of the “reference filter” from 2007, which allows comparison of the typical elemental patterns before and during the ^106^Ru episode. Generally, the elemental concentrations vary considerably when compared to the upper continental crust, spanning around two orders of magnitude. Some of these “outliers”, such as Sb, are generally associated with emissions caused by modern traffic (emission of abraded particles from brake pads) and urban environment in a large European city such as Vienna. This is not uncommon, as it has to be emphasized that only airborne particles have been sampled, which do not represent the bulk composition of the Earth’s crust.

For comparison of the determined elemental concentrations to other urban areas, the elemental mass fraction values from reference materials NIST SRM 1648a Urban Particulate Matter can be used. The comparison shows that there are no anomalies or uncommon occurrences of certain elements in unusual concentrations or ratios in urban living areas.

Please note that the the mass fractions of the reference filter from 2007 are constantly lower than from the ^106^Ru containing filter sample from 2017. It is apparent that the amount of filtered air has been much lower in 2007, although the actual amount remains unknown. This clearly indicates that a lower amount of particulate matter had been sampled in 2007 compared with 2017. The differences in the mass fraction range are mainly shifted to lower amounts with a factor of less than 10. In addition to the elements that have been displayed in Fig. 1, the following elements have been detected, but could not be quantified (due to a lack of certification values in the reference materials we used): gold, chlorine, bromine, osmium, and magnesium. No stable ruthenium isotopes were detected, because ruthenium is determined with low sensitivity in INAA. In addition, a spectral interference with activation products of barium raised the detection limit. With the exception of osmium, INAA did not reveal detectable amounts of other platinum group elements (Rh, Pd, Ir, and Pt), which are characterized by a similar chemical behavior like ruthenium. Although uranium is a common finding in INAA of geological materials [7–9], no traces have been detected in the filter.

### SEM and EDXS

**Fig. 2:**
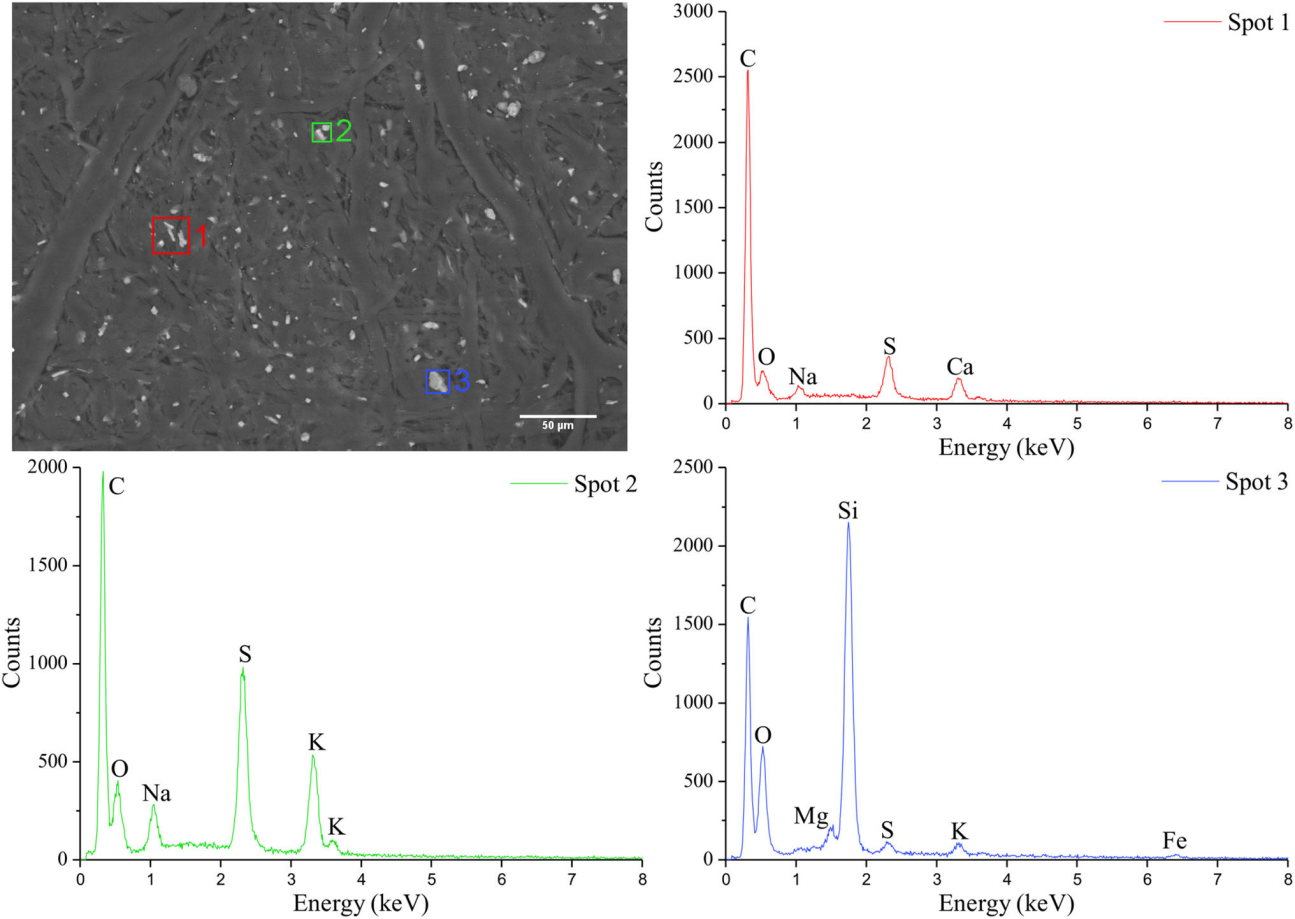
SEM pictures and EDXS spectra of three different spots found on the filter material

**Fig. 3:**
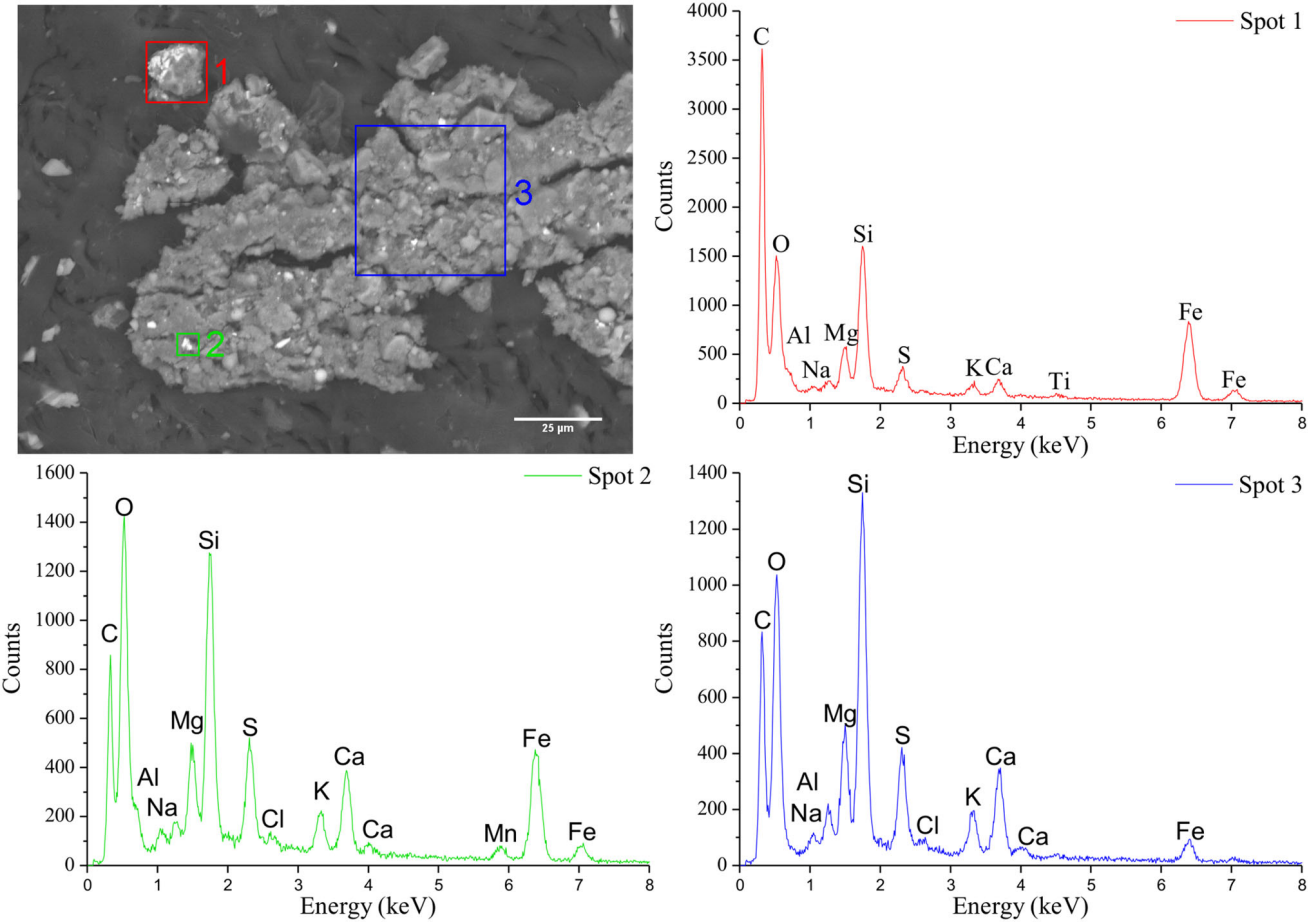
SEM pictures and EDXS spectra of three different spots on a larger agglomerate

Figures 2 and 3 show two SEM images of the ^106^Ru containing filter as well as three EDXS spectra of potentially interesting spots. The SEM images show the distribution of particles of various sizes that one would expect from an air filter in an urban area: the size distribution of dust particles ranges from a few µm up to a small amount of agglomerates with some hundreds µm in diameter. EDXS revealed that the majority of particles were made of light naturally abundant elements such as potassium, sodium, sulfur, oxygen, and silicon. Also, bigger agglomerates with heavier metals and elements such as manganese, iron, titanium, and chlorine were found. In summary, the patterns of particle sizes and elements within the particles reveal no uncommon characteristics. No abnormal particulate matter that could be indicative of an unusual phase carrying the ^106^Ru have been detected. This is in agreement with the observation that the ^106^Ru activities are evenly distributed on the filter surface, indicating a high degree of homogeneity.

## Conclusions

In this study, attempts have been undertaken to reveal possible anomalies in the chemical (including trace element) composition and the morphology of particles contained in an air filter that previously collected radioactive ^106^Ru in Vienna in fall 2017. By using the SEM/EDXS a general overview concerning the particle shape and major element composition was obtained. Neither particle shape nor composition revealed uncommon or unusual characteristics that may be indicative of an unusual carrier phase of the anthropogenic radionuclide ^106^Ru. No unusual heavy metal spots were found in the SEM imaging.

Instrumental neutron activation analysis was used to provide more comprehensive and thus more reliable chemical information on the stable element composition of the ^106^Ru-containing filter. By INAA, the chemical fingerprints of activatable major and trace elements of the ^106^Ru filter (2017) have been determined and compared to a filter that had been in operation 10 years ago in the same urban environment. This method generated values for 28 elements, however no uncommon element appearance or scale of mass fraction was determined. All values for 2017 are comparable with 2007. So no hint was found for a chemical/stable element irregularity or anomaly in the filtered, radioruthenium containing air from 2017 compared to 2007.
